# Oligonucleotide Microarray Analysis of Age-Related Gene Expression Profiles in Miniature Pigs

**DOI:** 10.1371/journal.pone.0019761

**Published:** 2011-05-13

**Authors:** Junko Takahashi, Masaki Misawa, Hitoshi Iwahashi

**Affiliations:** 1 National Metrology Institute of Japan, National Institute of Advanced Industrial Science and Technology, Tsukuba, Ibaraki, Japan; 2 Human Technology Research Institute, National Institute of Advanced Industrial Science and Technology, Tsukuba, Ibaraki, Japan; 3 Health Research Institute, National Institute of Advanced Industrial Science and Technology, Takamatsu, Kagawa, Japan; 4 Faculty of Applied Biological Sciences, Gifu University, Gifu, Japan; California State University Fullerton, United States of America

## Abstract

Miniature pigs are useful model animals for humans because they have similar anatomy and digestive physiology to humans and are easy to breed and handle. In this study, whole blood microarray analyses were conducted to evaluate variations of correlation among individuals and ages using specific pathogen-free (SPF) Clawn miniature pigs. Whole blood RNA is easy to handle compared to isolated white blood cell RNA and can be used for health and disease monitoring and animal control. In addition, whole blood is a heterogeneous mixture of subpopulation cells. Once a great change occurs in composition and expressing condition of subpopulations, their associated change will be reflected on whole blood RNA. From 12 to 30 weeks of age, fractions of lymphocytes, monocytes, neutrophils, eosinophils, and basophils in white blood cells showed insignificant differences with age as a result of ANOVA analysis. This study attempted to identify characteristics of age-related gene expression by taking into account the change in the number of expressed genes by age and similarities of gene expression intensity between individuals. As a result, the number of expressed genes was less in fetal stage and infancy period but increased with age, reaching a steady state of gene expression after 20 weeks of age. Variation in gene expression intensity within the same age was great in fetal stage and infancy period, but converged with age. The variation between 20 and 30 weeks of age was comparable to that among 30 weeks individuals. These results indicate that uniformity of laboratory animals is expected for miniature pigs after 20 weeks of age. Furthermore, a possibility was shown that whole blood RNA analysis is applicable to evaluation of physiological state.

## Introduction

Pigs are a useful model animals of humans because they have similar anatomy and digestive physiology to human [1]–[2]. In particular, miniature pigs are easier to breed and handle than other nonprimates, making them an optimal species for preclinical test [3]. Moreover, blood samples can be taken repeatedly and human medical devices such as endoscopes and MRI and CT scanners are also applicable. These advantages increasingly allow miniature pigs for laboratory animals, with recent progress in upgraded supply systems. In spite of some large-scale microarray studies on pigs, only a limited amount of fundamental data is available for pigs compared to other laboratory species [4]–[5]. In September 2003, the Swine Genome Sequencing Consortium (SGSC) was formed by industry, government, and academia, to promote pig genome sequencing under international coordination [6]. In November 2009, since the announcement of completed swine genome map by members of the SGSC, its research environment has been enhanced [7].

Microarray techniques allow to detect genome-wide perturbations during various treatments and to measure various responses by multitude of gene probes. Toxicogenomics, in which microarray techniques are specifically used in toxicology test, has been widely recognized as one of standard safety procedures for chemicals [8]–[10]. Gene expression microarrays have been used particularly for screening of genes involved in specific biological processes of interest, such as diseases or responses to environmental stimuli. Such experiments adopt the “healthy state” as a control, and identify highly expressed or suppressed genes. However, few studies deal with the features of gene expression and its variation at the “healthy state” to be influenced by species, age, sex, and individual variability. In measuring the state of disease and drug response, minimally invasive blood sampling, which allows for direct measurement of immune-responsive blood cells, excels other invasive biopsy techniques upon disease diagnostics and assessment of drug response, as well as health monitoring. Blood RNA contains an enormous amount of information on expression of messenger RNA and non coding functional RNA which remains without being translated into protein. Thus, blood RNA offers an opportunity to detect subtle change in physiological state. In this study, we conducted a series of whole blood microarray experiments to evaluate variations of correlation among individuals and ages using specific pathogen-free (SPF) miniature pigs.

Use of whole blood was intended on two accounts. First, we are aware that RNA expression and degradation are susceptible to artificial manipulation such as cell separation and extraction. Whole blood manipulation avoids this risk by using a RNA blood collection tube. Second, whole blood is a heterogeneous population of lymphocytes (monocytes, T-cells, and B-cells), granulocytes (neutrophils, eosinophils, and basophils), and platelets. It is expected that representative subpopulations in white blood cells may change depending on the condition of an individual. When a great alteration occurs in some subpopulations, whole blood will depart from the normal state of its age, because whole blood is a heterogeneous mixture of such subpopulations. We consider that it is particularly important to identify gene expression characteristics and variation of heterogeneous population of cells with age in whole blood.

The present microarray analysis was conducted from the following aspects. First analysis addresses the number and kind of expressed genes. Quantity and characteristics of expressed genes by age were examined. Second analysis deals with similarity among individuals based on the correlation coefficient. Variations among individuals of the same age group and that between different age groups were examined. These results offer age-related gene expression characteristics of miniature pigs when whole blood is employed.

## Results

### Characteristics of study subjects

Body weight change and hematological variation during breeding period are shown Table 1 and Table 2, respectively. One-way ANOVA analysis for age-related variations in red blood cell count (RBC), hemoglobin concentration (HGB), and hematocrit value (HCT) showed significant differences for both males and females. However, the mean corpuscular volume (MCV), mean corpuscular hemoglobin (MCH), and mean corpuscular hemoglobin concentration (MCHC) remained unchanged. Differences in platelet count (PLT) and fibrinogen level (Fbg) were significant only for females. Any significant differences were not observed for both males and females for Prothrombin time (PT), activated partial thromboplastin time (ATPP), and the white blood cell count (WBC). Similarly to humans, the ratio of lymphocytes to white blood cells increased with maturation from 16 to 30 weeks of age. However, its difference was statistically insignificant according to ANOVA analysis. From 12 to 30 weeks of age, the ratios of granulocytes (neutrophils, eosinophils, and basophils), lymphocytes, and monocytes to white blood cells were unchanged, and differences were also insignificant.

**Table 1:** Subject body weight results.

Sex	n	12 weeks	16 weeks	20 weeks	24 weeks	30 weeks	*P* †
Male	5	7.0±0.6	10.7±3.8	12.1±2.6	15.0±1.7	17.7±1.7	<0.001
Female	5	6.9±0.5	7.9±3.2	10.1±2.6	13.5±2.1	16.0±2.6	<0.001

Values are mean±SD.

†*P* values were calculated using one-way factorial ANOVA.

**Table 2:** Subject hematology results.

Hematological analysis	Sex	n	12 weeks	16 weeks	20 weeks	24 weeks	30 weeks	*P* †
**RBC, 10^4^/µL**	Male	5	742.7±72.6	858.0±97.7	894.8±55.8	919.0±21.0	866.2±24.5	<.05
	Female	5	727.0±20.2	886.6±62.2	921.2±64.5	901.4±46.1	838.4±44.2	<.001
**HGB, g/dL**	Male	5	14.9±1.6	16.4±1.2	17.3±0.6	18.3±0.4	17.7±0.3	<.001
	Female	5	14.9±0.4	17.5±0.8	18.0±0.9	18.4±1.1	17.5±0.6	<.001
**HCT, %**	Male	5	50.9±5.1	53.6±2.7	54.7±2.1	58.4±2.8	55.3±1.2	<.05
	Female	5	49.0±1.8	56.1±2.2	57.8±4.2	57.9±3.0	54.8±2.8	<.01
**MCV, fL**	Male	5	65.8±1.0	66.3±2.5	67.3±2.9	65.1±1.4	65.8±2.2	NS
**MCH, Pg**	Male	5	19.8±0.5	20.0±1.1	20.1±0.9	20.5±0.6	20.6±0.8	NS
**CHC, %**	Male	5	30.1±0.4	30.2±1.0	29.9±0.9	31.5±0.9	31.2±0.8	NS
**PLT, 10^4^/µl**	Male	5	21.3±0.4	31.6±10.8	18.1±4.4	25.0±8.6	24.9±5.1	NS
	Female	5	34.5±2.0	24.8±5.5	19.0±5.0	24.8±8.9	19.7±5.7	<.05
**PT, sec**	Male	5	13.8±3.2	15.5±0.3	16.5±0.9	15.9±0.7	16.1±0.6	NS
	Female	5	-	15.8±1.1	16.1±0.5	16.4±0.5	16.0±0.7	NS
**APTT, sec**	Male	5	<20	<20	<20	<20	<20	
	Female	5	<20	<20	<20	<20	<20	
**Fbg, mg/dl**	Male	5	171.3±36.9	185.8±93.8	169.4±39.4	158.6±9.0	147.8±34.2	NS
	Female	5	-	160.2±19.4	145.2±16.3	176.5±20.1	123.3±27.5	<.05
**WBC, 10^2^/µL**	Male	5	62.0±18.7	86.6±12.7	78.8±24.7	79.6±24.0	71.8±13.2	NS
	Female	5	66.0±23.4	74.0±13.7	78.0±18.7	72.4±10.4	61.8±11.3	NS
**Lymphocyte, %**	Male	5	34.8±12.1	45.2±7.4	44.6±9.3	36.8±6.9	33.6±7.6	NS
**Neutrophil, %**	Male	5	55.0±10.9	43.1±10.3	44.8±7.4	52.2±7.0	56.2±9.2	NS
**Eosinophil, %**	Male	5	3.8±2.2	3.1±1.4	3.0±1.9	5.0±2.7	4.6±1.7	NS
**Basophil, %**	Male	5	0.3±0.5	0.3±0.4	0.2±0.4	0.0±0.0	0.2±0.4	NS
**Monocyte, %**	Male	5	6.3±1.0	8.0±3.2	7.4±1.5	6.0±2.1	5.4±1.3	NS

Biochemical variables for miniature pigs during the experiment are shown. Values are mean±SD. RBC, red blood cell count; HGB, hemoglobin concentration; HCT, hematocrit value; MCV, mean corpuscular volume; MCH, mean corpuscular hemoglobin; MCHC, mean corpuscular hemoglobin concentration; PLT, blood platelet count; PT, prothrombin time; ATPP, activated partial thromboplastin time; Fbg, fibrinogen level; WBC, white blood cell count; and NS: not significant.

†*P* values were calculated using one-way factorial ANOVA.

### Microarray gene expression profiles - Number of expressed genes

To characterize the age-related gene expression in whole blood from miniature pigs, RNA analysis was conducted on bloods sampled from fetal stage, 12, 20, and 30 weeks subjects. Each RNA sample was analyzed by an Agilent #G2519F#20109 Porcine Gene Expression Microarray (44K) consisting of 43603 oligonucleotide probes.

Initially, we examined the change in the number of expressed genes to identify age-related characteristics. Microarray gene expressions were divided into two groups; “absent” and “present”, using flag indicators given by the scanner. Background level was determined from spot intensities outside the gene probing area. “Absent” was assigned to the spots whose intensities were less than the background level, while the rests were marked as “present.” Then each gene was judged as either “expressed” or “unexpressed” based on the number of “present” events. We defined a certain gene as “expressed” when “present” exceeds 75% out of replicated events. “A threshold of 75% was chosen by considering experimental deviation. Detailed explanation is given in the following part of Section “Materials and methods: Microarray data analysis.”

**Figure 1:**
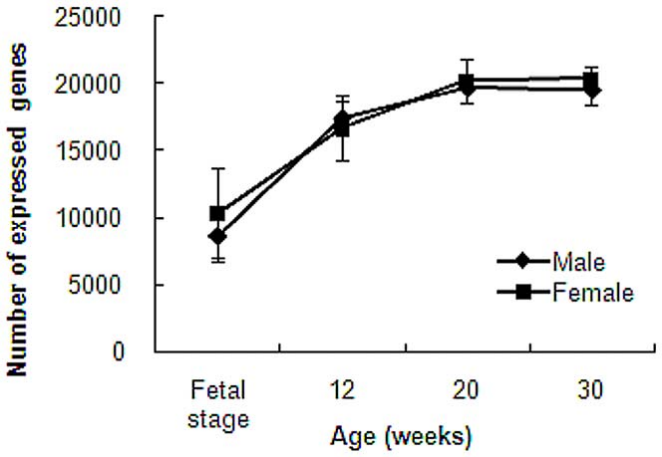
Number of genes expressed in whole blood of miniature pigs at different ages. In the graph, ♦ represents male and ▪ represents female. Values are means±SD.

The number of expressed genes increased with age for both male and female subjects, as shown in Figure 1. Expressed genes for male and female were analyzed by one-way factorial ANOVA. Then Tukey-Kramer's method was applied only to significant groups. Differences between age groups (fetal stage, 12, 20, and 30 weeks of age) were significant for male, female, and mixed subjects of male and female. A Tukey-Kramer's multiple comparisons test revealed that differences between fetal stage and other age groups were statistically significant (p<0.001) for both male and female. Also, differences were significant (P<0.05) between 12 and 30 weeks females.

### Microarray gene expression profiles – Correlation of gene expression

**Figure 2:**
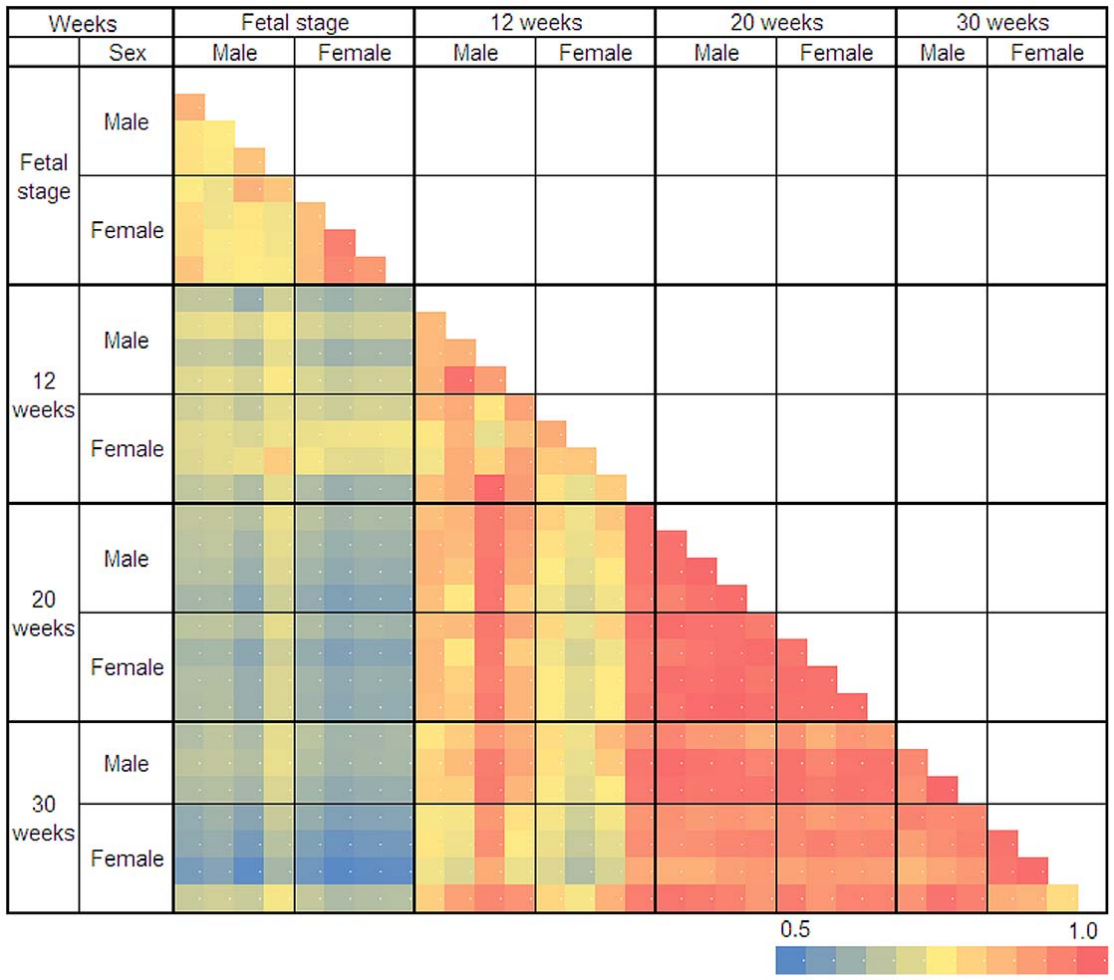
Correlation matrix of age-related gene expression. This color-coded correlation matrix illustrates pairwise correlations between the levels of gene expression in individuals. Probe sets with normalized signals (log-transformed and scaled) were used to calculate correlations between 31 arrays using Pearson correlation coefficient; signals flagged as “absent” were excluded.

We evaluated variations in correlation coefficients among individuals of the same age and different age groups. Pearson correlation coefficient was used for correlation analysis. Correlation coefficients for a total of 31 microarrays were obtained in normalized signals log-scale after excluding “absent” spots. A color-coded pairwise correlation matrix is shown in Figure 2. The color scale at the bottom indicates correlation strength.

Table 3 shows the average correlation coefficients among individuals within the same age group, while Table 4 presents those between different age groups. Pearson correlation coefficient between two individuals was calculated from the spot intensities. The number of spots varied depending on selected pairs because “absent” spots were excluded from the analysis. The numbers of analyzed spots for the same aged pair were 7041±1703, 4728±1425, 18534±517, and 18160±1320, for fetal-stage, 12 weeks, 20 weeks, and 30 weeks, respectively. P-value was calculated from t-value, which was obtained from the Pearson correlation coefficient r and the number of “present” spots n between the paired individuals. The maximum p-value in our study is expected for r = 0.42 with 8691 spots. The p-value may be infinitely small. These analyses led us to use r instead of p-value as an indicator of significance.

**Table 3:** Summary of age-related correlation coefficients within the same age groups.

Age group	Sex	N	Correlation coefficient
**Fetal stage**	Male	4	0.87±0.02
	Female	4	0.93±0.03
	Male and Female	8	0.87±0.04
**12 weeks**	Male	4	0.93±0.03
	Female	4	0.88±0.04
	Male and Female	8	0.90±0.04
**20 weeks**	Male	4	0.98±0.01
	Female	4	0.98±0.00
	Male and Female	8	0.98±0.01
**30 weeks**	Male	3	0.96±0.02
	Female	4	0.94±0.05
	Male and Female	7	0.95±0.03

Average correlation coefficients calculated within the groups of males, groups of females, groups of males and females of the same age. Values are means±SD.

**Table 4:** Summary of age-related correlation coefficients between different age groups.

Age group	Sex	Correlation coefficient
**Fetal stage vs. 12 weeks**	Male and Female	0.73±0.07
**Fetal stage vs. 20 weeks**	Male and Female	0.63±0.07
**Fetal stage vs. 30 weeks**	Male and Female	0.61±0.10
**12 weeks vs. 20 weeks**	Male and Female	0.89±0.06
**12 weeks vs. 30 weeks**	Male and Female	0.87±0.07
**20 weeks vs. 30 weeks**	Male and Female	0.95±0.02

Values are means±SD.

The average correlation coefficient within the same age group were 0.87±0.04 ,0.93±0.03, 0.93±0.03, 0.88±0.04, 0.98±0.01, 0.98±0.00 ,0.96±0.02, 0.94±0.05 for fetal sage males, fetal stage females, 12 weeks males, 12 weeks females, 20 weeks males, 20 weeks females, 30 weeks males, and 30 weeks females, respectively. Variations in gene expression were greater for younger subjects, but it diminished with age while generating resembling expression patterns (Table 3). Correlation coefficient within 30 weeks age group was slightly smaller than that within 20 weeks age group. However, this difference is still within the standard deviation and is smaller than other distant age groups. Whole blood gene expression was analyzed also for 40 weeks age group (males, n = 4) and 65 weeks age group (females, n = 4). The corresponding correlation coefficients were 0.96±0.02 and 0.97±0.01, respectively. Similar gene expressions to 30 weeks age group were observed. Although the data set is not complete for 40 and 65 weeks groups, this data supports a steady gene expression after maturity.

Next, we analyzed correlation between different age groups. In Table 4, “20 vs. 30 weeks” represents the average correlation coefficient between 20 weeks age group and 30 weeks age group individuals. Correlation coefficients became smaller between “fetal vs. 12 weeks” and “12 vs. 20 weeks”. This indicates that correlation between 20 and 30 weeks age group is higher than any other distant age groups. In addition, correlation coefficient between 20 weeks and 30 weeks age group was 0.95±0.03. This is similar to that within 30 weeks age group (0.95±0.02), indicating that gene expression pattern at 20 weeks resembles that at 30 weeks. The number and correlation of expressed genes were dependent on age but less dependent on sex (Figure 1 and Figure 2). Average correlation coefficients between males and females at the same age were greater than that between different age groups of the same sex (Table 3 and Table 4). Then we assumed that these variations were mainly due to progress in age, and focused on age difference in the subsequent analyses.

### Classification of genes depending on the status of age-related expression

**Figure 3:**
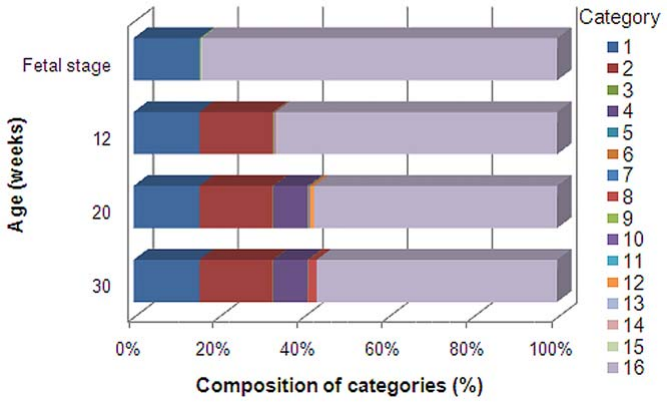
Ratios of categories for groups of the same age. The ratios of the genes in each category were calculated for groups in the fetal stage and at 12, 20, and 30 weeks of age. Categories are defined in Table 5.

All spots on the microarray were divided into 16 categories as shown Table 5 after assigning “1” for expressed genes and “0” for unexpressed genes. Here, definitions of “expressed” and “unexpressed” are described in “Materials and methods.” Category 1 consists of a total of 6,763 genes expressed in the fetal stage, 12, 20, and 30 weeks of age. Category 2 consists of a total of 7,564 genes expressed at 12, 20, and 30 weeks of age. Category 4 consists of a total of 3,547 genes expressed after 20 weeks of age. Category 8 consists of a total of 827 genes expressed after 30 weeks of age. Sum of the genes expressed at certain age and those unexpressed (Categories 3, 5, 6, 7, 9, 10, 11, 12, 13, 14, and 15) was 1,051. Its fraction was 5.6% of 18,701 genes (Categories 1, 2, 4, and 8) expressing constantly once they appeared. Category 16 consists of genes unexpressed throughout the breeding period. Figure 3 shows the ratio of the genes belonging to each category.

**Table 5:** Genes classified into 16 categories according to the status of age-related expression.

Category	Fetal stage	12 weeks	20 weeks	30 weeks	Number of genes	Definition
1	1	1	1	1	6763	genes expressed from fetal stage to 30 weeks
2	0	1	1	1	7564	genes expressed from 12 to 30 weeks
3	1	0	1	1	49	
4	0	0	1	1	3547	genes expressed from 20 to 30 weeks
5	1	1	0	1	14	
6	0	1	0	1	80	
7	1	0	0	1	7	
8	0	0	0	1	827	genes expressed at 30 weeks
9	1	1	1	0	73	
10	0	1	1	0	124	
11	1	0	1	0	29	
12	0	0	1	0	428	genes expressed at 20 weeks
13	1	1	0	0	16	
14	0	1	0	0	147	genes expressed at 12 weeks
15	1	0	0	0	84	genes expressed in fetal stage
16	0	0	0	0	23851	genes not expressed from fetal stage to 30 weeks

Depending on the status of expression, all spots on the microarray can be divided into 16 categories. Here, “1” represents an expressed gene and “0” represents an unexpressed gene.

### Characteristics of expressed genes in different categories - Variations of the correlation coefficients

To estimate the cause of variation in gene expression during infancy period, correlation coefficients between same-aged individuals were calculated for all spots and those belonging to Categories 1, 2, 4, and 8 (Table 6). Correlation coefficient for genes in Category 1 was almost the same with that for all genes. Statistical significance by the t-test was negative between all genes and those in Category 1 at any periods of age. Thus, enhanced variation in gene expression at infancy period may be caused by individual fluctuation, rather than by gene types.

**Table 6:** Age-related correlation coefficients for each gene set by classification.

		n	All spots	Category 1	Category 2	Category 4	Category 8
**Number of genes**			43603	6763	7564	3547	827
**Age group**	Fetal stage	8	0.87±0.04	0.88±0.04	-	-	-
	12 weeks	8	0.90±0.04	0.89±0.05	0.77±0.10	-	-
	20 weeks	8	0.98±0.01	0.98±0.01	0.96±0.01	0.80±0.04	-
	30 weeks	7	0.95±0.03	0.94±0.03	0.92±0.04	0.72±0.11	0.29±0.21

Number of genes and correlation coefficients in each category. The average correlation coefficients were calculated between individuals for all spots and gene sets for Categories 1, 2, 4, and 8 within the same age group.

### Assigning known functions to gene expression - Gene ontology annotation

To characterize gene expression in each category, TC Annotator List (Porcine version 14.0 3-11-10) was downloaded from the TIGR gene Indices. TC Annotator List includes the gene number and the GO terms. Out of 43,603 probes in the Agilent porcine microarray (#G2519F#20109), 6,019 genes bear GO annotation. Microarray cDNA probes were classified by GO terms of “biological processes”. Out of all genes, fraction in Categories 1, 2, 4, 8, and 16 were 31%, 20%, 8%, 2%, and 38% respectively.

**Figure 4:**
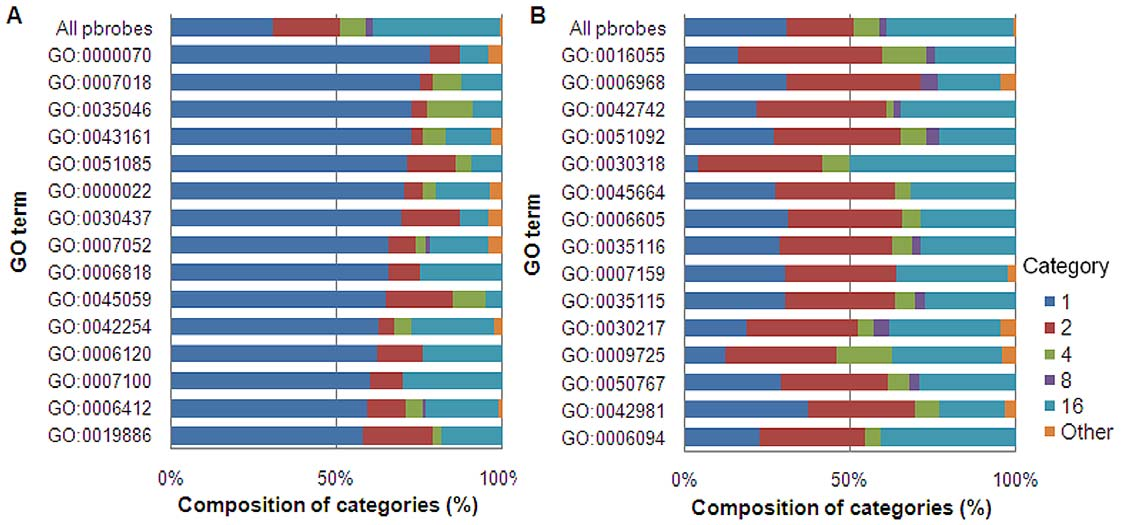
Proportion of genes, by category, for genes with specific GO terms relating to biological processes. (A) Category 1. (B) Category 2. The ratio of the genes in each category was calculated for selected GO terms. Categories are defined in Table 5.

Then the difference in gene expression between all spots and those in 4 categories (Categories 1, 2, 4, and 8) was examined. Among more than 20 genes classified under “biological processes” in GO term, dominant ones in Category 1 and 2 are shown in Figure 4. A negligible difference was seen for gene in Categories 4 and 8. In Table 7 and Table 8, GO Accession in Figure 4 is related to GO terms together with the number of genes. GO groups dominantly expressed in Category 1 relates to mitosis (GO:0000070, GO:0000022, GO:0007052, and GO:0007100) and to immune (GO:0043161, GO:0045059, GO:0019886), while those highly expressed in Category 2 related to cellular defense and regulation.

**Table 7:** Predominant genes with GO terms related to biological processes in category 1.

Accession	Number of genes	GO term
GO:0000070	23	mitotic sister chromatid segregation
GO:0007018	24	microtubule-based movement
GO:0035046	22	pronuclear migration
GO:0043161	29	proteasomal ubiquitin-dependent protein catabolic process
GO:0051085	21	chaperone mediated protein folding requiring cofactor
GO:0000022	54	mitotic spindle elongation
GO:0030437	23	ascospore formation
GO:0007052	73	mitotic spindle organization
GO:0006818	32	hydrogen transport
GO:0045059	20	positive thymic T cell selection
GO:0042254	40	ribosome biogenesis
GO:0006120	29	mitochondrial electron transport, NADH to ubiquinone
GO:0007100	20	mitotic centrosome separation
GO:0006412	253	Translation
GO:0019886	43	antigen processing and presentation of exogenous peptide antigen via MHC class II

GO terms and the number of genes in the Agilent porcine microarray for each Accession are shown in Figure 4A.

**Table 8:** Predominant genes with GO terms related to biological processes in category 2.

Accession	Number of genes	GO term
GO:0016055	37	Wnt receptor signaling pathway
GO:0006968	42	cellular defense response
GO:0042742	46	defense response to bacterium
GO:0051092	26	positive regulation of NF-kappaB transcription factor activity
GO:0030318	24	melanocyte differentiation
GO:0045664	22	regulation of neuron differentiation
GO:0006605	35	protein targeting
GO:0035116	35	embryonic hindlimb morphogenesis
GO:0007159	36	leukocyte cell-cell adhesion
GO:0035115	33	embryonic forelimb morphogenesis
GO:0030217	21	T cell differentiation
GO:0009725	24	response to hormone stimulus
GO:0050767	31	regulation of neurogenesis
GO:0042981	56	regulation of apoptosis
GO:0006094	22	Gluconeogenesis

GO terms and the number of genes in the Agilent porcine microarray for each Accession are shown in Figure 4B.

Table 9 shows correlation coefficients within the same age group for genes bearing GO terms such as neutrophil, monocyte, and platelet. Those under “neutrophil” were 0.95±0.03, 0.93±0.03, 0.98±0.01, and 0.97±0.02 for fetal stage, 12 weeks, 20 weeks, and 30 weeks age groups, respectively. On the other hand, those under “platelet” were 0.96±0.02, 0.94±0.04, 0.99±0.01, and 0.97±0.02, respectively, maintaining high correlation regardless of age. Correlation coefficients under “monocyte” varied greater than the former, and even greater under “metabolic process.”

**Table 9:** Age effect on correlation coefficients for GO term biological processes.

		n	Spots with GO term	Neutrophil	Monocyte	platelet	Metabolic processes
**Number of genes**			6019	57	50	36	1020
**Age group**	Fetal stage	8	0.90±0.04	0.95±0.03	0.92±0.06	0.96±0.02	0.89±0.05
	12 weeks	8	0.93±0.03	0.93±0.03	0.92±0.04	0.94±0.04	0.93±0.03
	20 weeks	8	0.99±0.01	0.98±0.01	0.98±0.01	0.99±0.01	0.98±0.01
	30 weeks	7	0.96±0.02	0.97±0.02	0.95±0.03	0.97±0.02	0.96±0.02

Given in the above table are predominant biological processes defined by Gene Ontology.

### Age-related changes in gene expression levels for the immune system

Next, expression intensity of immunity gene was examined. “Biological Process” contains immune system process (GO accession 0002736) attaching 11 subsets below (Table 10). Antigen processing and presentation (GO:0019882) and T-cell selection (GO:0045058) holds large amount of Category 1 genes constantly expressing since fetal stage. Many of Category 2 were under leukocyte homeostasis (GO:0001776) and lymphocyte costimulation (GO:31924). These genes were expressed constantly after 12 weeks.

**Table 10:** GO terms for a subset of GO:0002376 immune system processes.

	Number of genes in each category						
Accession	All spots	Category1	Category2	Category4	Category8	Category16	GO term
GO:0001776	10	1	5	1	2	1	leukocyte homeostasis
GO:0002200	9	0	2	0	0	5	somatic diversification of immune receptors
GO:0002252	60	15	16	3	0	22	immune effector process
GO:0002253	62	23	25	5	2	31	activation of immune response
GO:0002507	1	0	1	0	0	0	tolerance induction
GO:0006955	356	102	63	18	6	102	immune response
GO:0019882	140	31	11	3	1	15	antigen processing and presentation
GO:0031294	4	0	2	0	0	1	Lymphocyte costimulation
GO:0045058	44	22	7	3	0	9	T cell selection
GO:0045321	125	21	29	4	4	34	leukocyte activation
GO:0050900	76	23	18	1	0	23	leukocyte migration

The number of genes in selected categories was counted for each GO term. The GO term and the number of genes in the Agilent porcine microarray are also shown in the table. Categories are defined in Table 5.

**Figure 5:**
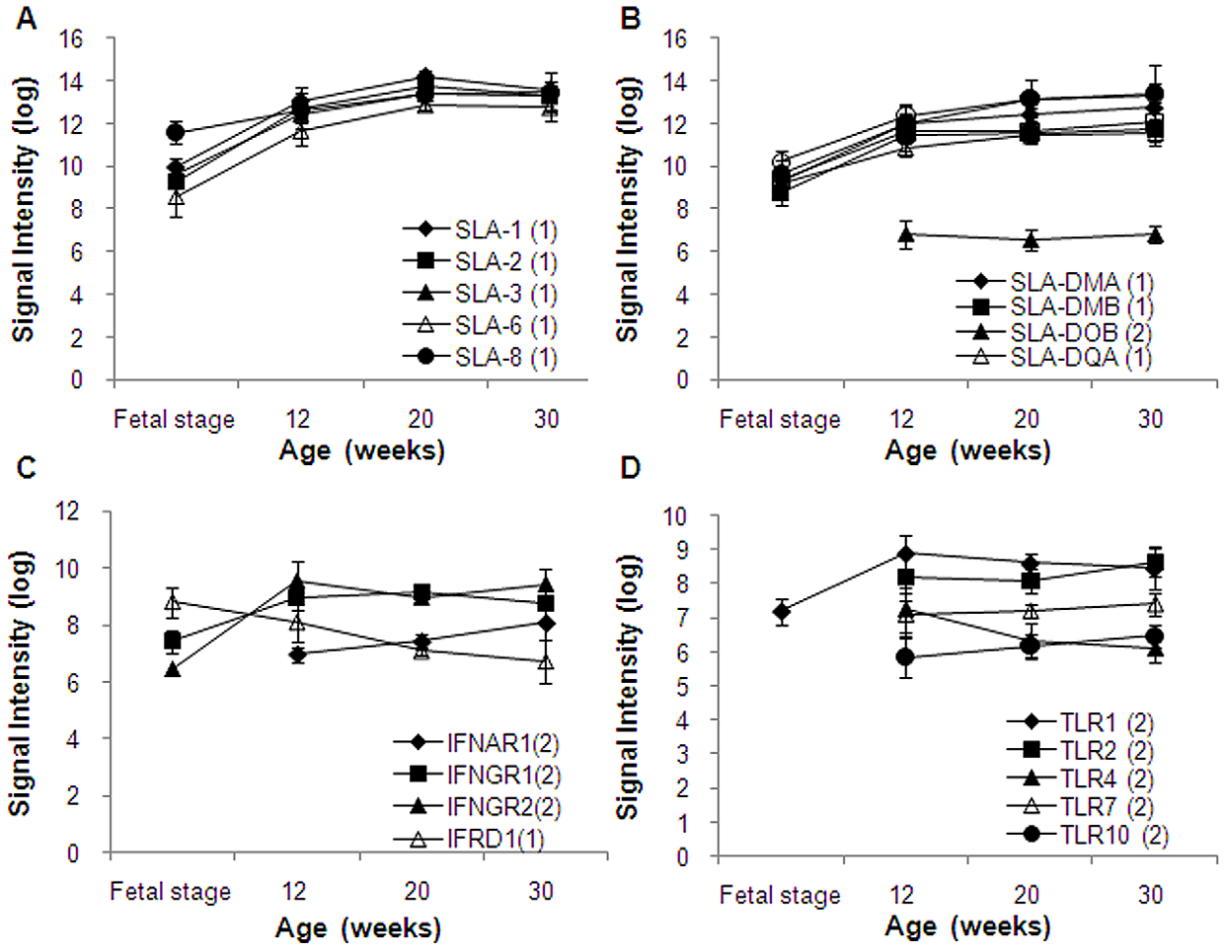
Signal intensity of major histocompatibility complex (MHC) genes. (A) Swine leukocyte antigens (SLA) classical class I genes. (B) Swine leukocyte antigens (SLA) classical class II genes. (C) Interferon receptor genes. (D) Toll-like receptor (TLR) genes. Signal intensities were normalized using quantile normalization and log-transformed after excluded signals flagged as “absent.” The category numbers are shown in graph legends. Genes in Categories 1, 2, and 4 are shown in the graph.

Antigen processing and presentation (GO:0019882) and T cell selection (GO0045058) include the major histocompatibility complex (MHC) genes. By presenting antigens, MHC is involved in elimination of bacterial or viral pathogen, rejection of cancer cells, and rejective response on organ transplantation. Also MHC is indispensable in the immune system. Swine leukocyte antigens (SLA) are important immunogens for humoral responses and important mediators of the cellular immune responses through both direct and indirect presentation of peptides to T-cells [11]. SLA includes 6 of classical class I genes (SLA-1, SLA-2, SLA-3, SLA-6, SLA-7, and SLA-8) and 8 of classical class II genes (SLA-DMA, SLA-DMB, SLA-DOA, SLA-DOB1, SLA-DQA, SLA-DQB1, SLA-DRA, and SLA-DRB1) [12]–[13]. SLA class II lacks DPA1, DPB1, DRB3, DRB4, and DRB 5 in humans. On the Agilent porcine microarray, all of SLA genes except DOA are mounted on 28 spots. Among these, 11 SLA genes fell under Category 1, 1 fell under Category 2, and 1 fell under Category 8. Expression of SLA classical class I and class II genes are shown in Figure 5A and 5B, respectively. Both genes expressed in fatal stage, 12, 20, and 30 weeks in an increased manner by age.

The Agilent porcine microarray had 7 probes with 7 types of interferon and 7 probes for 4 types of interferon receptors. All of 7 interferon genes fell under Category 16. Normally these genes remain unexpressed but expressed upon necessity. In contrast, 1 type of interferon receptor gene fell under Category 1, 3 fell under Category 2, and were expressed until 12 weeks of age. Their signal intensities stayed at constant levels after 12 weeks (Figure 5C).

Toll-like receptors (TLRs) are the principal pattern recognition receptors. With this innate immunity, the first immune response is mediated into reserved foreign patterns on recognition. TLRs recognize reserved molecular patterns, start rapid response to protect the host upon infection, and produce signals, such as cytokines and co-stimulatory molecules to activate the adaptive immune system [14]–[15]. Regulation of the TLR signaling cascade is important for inflammatory responses, innate host defense, and adaptive immune responses [16]–[17]. Most mammalian species are estimated to have between 10 and 15 types of TLRs. The Agilent porcine microarray has 10 types of TLRs probes. Among these TLRs, 5 of TLR genes fell under Category 2 (expressed until 12 weeks of age), 1 under Category 8, and 4 under Category 16. Their signal intensities remained constant after 12 weeks of age (Figure 5D).

## Discussion

This study aimed to evaluate the “healthy state” gene expression by whole blood microarray analysis of miniature pigs at different age groups up to 30 weeks. Results of our gene expression analysis can be applied to health management of laboratory animals, and eventually, to health monitoring of humans.

Use of whole blood was intended on two accounts. First, we are aware that RNA expression and degradation is susceptible to artificial manipulation such as cell separation and extraction. The whole blood manipulation avoids this risk, unlike dealing with extracted white blood cells. In addition, whole-blood RNA can be stabilized immediately by using RNA blood sampling tube such as PAXgene. This avoids the cell separation process after sampling and minimizes the possibility of RNA denaturation. Usually, peripheral blood mononuclear cells (PBMCs) separation employs the difference of specific gravity between other blood components, which should be followed immediately after the blood sampling. Such manipulation requires a skilled operator to reduce the influence of separation procedures on gene expression. Second, the whole blood is a heterogeneous population of cells. One can expect that representative subpopulations in white blood cells may vary depending on the health condition of an individual. When a great alteration occurs in some subpopulations, the whole blood may also depart from the normal state of its age, because whole blood is a heterogeneous mixture of such subpopulations. Therefore, identification of gene expression characteristics and age-related variation in subpopulations in whole blood are essential issues.

A major drawback in whole blood RNA manipulation includes a decrease in detection sensitivity caused by excessive globin mRNA. A previous study showed that globin elimination from whole blood RNA lead to alteration in original gene expression profiles [18]. Although a hybridization method is developed for globin elimination from total RNA preparations, such method is dedicated for humans, rats, and mice, and does not guarantee its validity for pigs. Therefore, in this study, we declined the globin elimination process because our intention is to develop a simplified mRNA analysis method capable of detecting variation of gene expression with moderate sensitivity.

Whole blood contains a variety of cell types as red blood cells, granulocytes, lymphocytes, and platelets. Most of the nucleated cells in blood are white blood cells such as neutrophils, T-cells, B-cells, and monocytes. The intraclass correlation, a measure of signal to noise ratio, of the difference between *Staphylococcus* enterotoxin B-stimulated and unstimulated blood from healthy subjects was significantly higher in leukocyte-derived samples the in whole blood, suggest that the method of RNA isolation of from whole blood is critical variable in blood RNA assay [19]. Although PBMCs do not contain neutrophils, eosinophils, basophils, nor platelets, Min et al. reported a highly correlated result (r^2^ = 0.85) for 8273 genes expressed in both whole blood RNA and PBMC RNA samples [20]. Other workers conducted a large scale genome-wide expression analysis of white blood cells subpopulations. This study indicates that correlation coefficients for T-cells and monocytes among different healthy subjects were 0.98±0.01 and 0.97±0.01, respectively. However, for the same subjects (n = 5), correlation coefficients between T-cells and monocytes was 0.88±0.01, indicating varied correlation between white blood cells subpopulations [21]. The number of white blood cells in humans is known to decrease steadily from infancy to adulthood, and its composition (i.e. lymphocytes, granulocytes) also changes with age [22]. In this study, hematological data, of the fetal stage was unavailable because the amount of collected blood was insufficient for the analysis. From 12 to 30 weeks of age, ANOVA analysis indicated no significant differences in the fractions of lymphocytes, neutrophils, eosinophils, basophils, and monocytes. In addition, these compositions were almost equal to those in human adults. The above result suggests that RNA originated from white blood cells and composition of subpopulations within the whole blood will remain unchanged from 12 to 30 weeks of age.

As a result of gene expression analysis based on whole blood RNA, the number of expressed genes was less in fetal stage and infancy period but increased with age, presenting a decreased rate of gene expression after 20 weeks of age. Variation in gene expression intensity within the same age was great in fetal stage and infancy period, but decreased with age. The variation between 20 and 30 weeks of age was comparable to that among individuals. If we assume that the whole blood RNA is originated from blood cells, variation of gene expression in infancy period is attributed to that of blood cells themselves. Correlation coefficient analysis of genes expressed constantly from fetal stage to 30 weeks (Category 1) also agrees with this assumption. These results indicate that uniformity of laboratory animals is expected for miniature pigs after 20 weeks of age. The organs and reproductive function of the Clawn miniature pigs become mature at about 26 weeks. Physiological state will become stable from 20 weeks and gene expression will reach a steady state.

The expressed genes were classified into those expressed constantly and those emerged from certain age. Their functions were analyzed by Gene Ontology. As a result, we found that mitosis and immunity genes were distinctively expressed throughout the period from fetus to 30 weeks old, while defense and control genes began to express from 12 weeks old. The number of expressed genes converged after 20 weeks old. Functions for those expressed at 20 and 30 weeks old were unavailable due to limited number of porcine gene annotations.

Then, we examined variation of correlation coefficients for genes bearing GO terms such as neutrophil, monocyte, and platelet. Variation under “neutrophil” and “platelet” within the same age group was small from fetal stage to 30 weeks. On the other hand, that under “monocyte” became greater than the former, and even greater under “metabolic process.” These results suggest that variation of gene expression during fatal stage and infant period is caused by cell activity, rather than by blood cell subpopulation functions. Thus, whole blood RNAs is useful to monitor health condition of an individual since they reflect the status of white blood cell subpopulations.

In clinical use of whole blood RNA analysis, we need to validate that the state of immune-related gene expression in whole blood reflects the state of the immune system. Our Gene Ontology analysis showed that immune-related genes accounted for 87% of total expressed genes at 12 weeks, and 94% at 20 weeks. Our results indicated that MHC class I and class II genes were constantly expressed from fetal stage to 7 weeks and that the gene expression rate increased with age until 20 weeks, reaching a steady state afterwards. This tendency agrees with that noted in previous studies [23]–[28]. We also note that TLRs stated to form at fatal stage and maintained its expression level after birth, which agrees with previous reports [29]–[30]. Expression behavior of MHC and TLR gene groups detected by the whole blood RNA analysis suggest that age-related changes in the number of expressed genes and their expression characterize the features of its age. Because the immune system of the Clawn miniature pigs becomes mature after 20 weeks, immune-related genes will contribute to the amount and correlation of gene expression between adult individuals.

In this study, we proved that whole blood RNA analysis can be used in practical applications by considering variations of gene expression by individual specificity and age in miniature pigs. Further study on gene expression profiles for the early stages of disease and physiological state under stress will contribute to the promotion of xenotransplantation in miniture pigs, as well as to standardization of their breeding method. The blood RNA diagnostics under development can eventually be applied to human health monitoring.

## Materials and Methods

### Animals

Five males and five females of 12 week old Clawn miniature pigs were housed individually in cages of 1.5 m^2^ at the SPF facility of the breeder (Japan Farm Co., Ltd, Kagoshima, Japan) for 18 weeks. Mean body weights of males and females at the beginning of the experiment were 7.0 kg and 6.9 kg respectively. During this period, all animals were fed with 450 g/day standard dry feed (Kodakara73, Marubeni Nisshin Feed Co., Ltd., Tokyo Japan) with free access to water. Fetuses were taken out from their mothers on days 77 to the 84 days of the pregnancy by a Caesarean section. The unborn baby's sex was determined based on the shape of the vulva.

### Hematology and clinical chemistries

All blood samples were collected from the superior vena cava at 12, 16, 20, 24, and 30 weeks of age. Blood (EDTA), plasma (EDTA) and serum samples for hematology and biochemical tests were collected 24 hours after fasting. Hematology and biochemical tests were conducted by Clinical Pathology Laboratory, Inc. (http://www.patho.co.jp/index.html) (Kagoshima, Japan) using standard clinical methods.

### MIAME compliance and data availability

The microarray experiments described in this manuscript are MIAME compliant and the raw data has been deposited in the Gene Expression Omnibus (GEO) database (Accession number GSE25393).

### Preparation of samples and microarray assay

Blood samples for microarray analysis were collected from each subject in PAXgene™ tubes (Qiagen/BD GmbH, UK), incubated at room temperature for 4 hours for RNA stabilization, and then stored at −80°C. RNA was extracted from whole blood using the PAXgene™ Blood RNA System Kit (Qiagen GmbH, Germany) following the manufacturer's guidelines. Quality of the purified RNA was verified on an Agilent® 2100 Bioanalyzer (Agilent Technologies, Santa Clara, CA). RNA concentrations were determined using a NanoDrop® ND-1000 spectrophotometer (NanoDrop Technologies, Wilmington, DE). cRNA labeled with fluorescent Cyanine 3-CTP was used for hybridization onto porcine oligo microarray slides (#G2519F#20109, Agilent Technologies) containing 43,603 oligonucleotide probes at 65°C for 17 h. Hybridized microarray slides were washed according to the manufacturer's instructions and were scanned with an Agilent DNA Microarray Scanner (#G2565BA, Agilent Technologies) at 5-µm resolution. The scanned images were analyzed numerically using Agilent Feature Extraction Software version 9.5.3.1. (Agilent Technologies)

### Microarray data analysis

Normalized data using quantile normalization were analyzed using GeneSpring GX software version 10.0.1 (Agilent Technologies). The Gene Ontology Database (http://www.geneontology.org/) was used to functionally categorize gene expression profiles. GO terms were obtained from the TIGR pig gene indices, Porcine version 14.0 3-11-10 (http://compbio.dfci.harvard.edu/cgi-bin/tgi/gimain.pl?gudb=pig).

In microarray data analysis, we particularly focused on the variation of age-related gene expressions profiles. Initially, microarray spots of interest were divided into two groups; “absent” and “present”, using the flag values given by the scanner. Background level was determined from the spots outside the gene probing area. “Absent” was assigned to the spots whose signal intensity was less than background level, while the rests were marked as “present.” Scanned images of microarray slides were analyzed numerically using the Agilent Feature Extraction Software. The spot information data file includes the following flag values: “feature is not positive and significant,” “feature is not uniform,” “feature is not above background,” “feature is saturated,” and “feature is a population outlier.” Microarray signals flagged as “not positive and significant” or as “not above significant” were defined as “absent.” To be more specific, “signal feature is positive and significant” means that the mean signal of a feature is greater than the corresponding background and that this difference is significant. “Feature is not above background” means that a feature has background-subtracted signals greater than 2.6 SD. The values greater than 2.6 SD fall into a 99% confidence interval. On the other hand, “present” includes flag values as “positive,” “feature is not uniform,” “feature is saturated,” and “feature is a population outlier.”

Then each gene was judged either “expressed” or “unexpressed,” based on the criterion that “present” occupies more than 75% in a series of replicated experiments. Its fluctuation is caused by the experimental errors, individual variation, and sensitivity difference in microarray. We chose the threshold value of 75% as a result of the Fisher's exact probability test for the number of samples = 8, which is equal to the number of individuals in a group of the same age in our study.

### Statistical analysis

Numeric values are presented as mean±standard deviation. Continuous variables were analyzed by one-way factorial ANOVA followed by Tukey-Kramer multiple comparisons test for multiple groups. Correlations were considered to be statistically significant when ANOVA test among all groups and t-test between 2 groups should p<0.05. To identify similarities in gene expression among individuals of the same age or between different age group, Pearson correlation coefficients were calculated after excluding unexpressed genes in each array data.

### Ethical considerations

All experimental protocols were approved by the Committee for the Care and Use of Experimental Animals at AIST (Permit Number: 2009-055A).
